# Difference of Bacterial Community Structure in the Meadow, Maize, and Continuous Cropped Alfalfa in Northeast China

**DOI:** 10.3389/fmicb.2022.794848

**Published:** 2022-03-31

**Authors:** Zhao Yang, Yanxia Xu, Hong Li, Shasha Li, Xiaolong Wang, Hua Chai

**Affiliations:** Branch of Animal Husbandry and Veterinary, Heilongjiang Academy of Agricultural Sciences, Qiqihar, China

**Keywords:** meadow, maize, continuous cropping alfalfa, bacterial structure, network

## Abstract

Maize and alfalfa (*Medicago sativa* L.) have been used extensively in the animal husbandry to compensate for the lack of livestock and fodder yields in the chilly northeast of China. Little is known, however, about the impact on soil characteristics of consecutive plantings in various crops and alfalfa. In this research, the soil characteristics, bacterial community diversity, and structure of the meadow, maize, and alfalfa continuous cropping fields (i.e., 6, 10, 14, 20, and 30 years) were measured. The results showed that maize cropping and continuous cropping of alfalfa increased the soil bacterial alpha diversity compared with meadow cropping, and alpha diversity of alfalfa increased with the continuous planting years. Soil pH, total phosphorus (TP), available P, total potassium (TK), and nitrate nitrogen (NO_3_^–^) content were soil variables significantly impacting the structure of soil bacterial communities in different plant types and different alfalfa continuous cropping systems. In addition, the relative abundance of some beneficial microbial species, such as *Arthrobacter* and *Gaiellales*, in the cropping maize and continuous cropping of alfalfa was much higher than that in the meadow field. Moreover, the networks differ among different plant types, and also differ among different continuous cropping years of alfalfa, and topologies of the networks suggested that continuous planting of alfalfa promotes cooperation between bacteria, which facilitates the long growth of alfalfa and is beneficial to the soil.

## Introduction

Alfalfa (*Medicago sativa* L.) is one of the most significant perennial herbaceous legume fodder in the world that is widely grown in many countries and contributes significantly to the development of agriculture and livestock (Han et al., 2005; Raiesi, 2007; Li and Huang, 2008). In China, alfalfa grows mainly in the arid and semi-arid soil of northern China and is grown on more than 4,000 ha per year (Zhang et al., 2016). The Northeastern portion of China is an agro-pastoral area with longer winters (Chen et al., 2013). As a result, animal feed in this region is almost entirely dependent on summer pasture and winter silage, and therefore, alfalfa can alleviate forage shortages for cattle in the winter in the Northeast with its more complete nutrition and high yield (Su, 2007; Chen et al., 2013). Thus, alfalfa has been continuously grown on a large scale in northeastern China to meet the winter demand for forage and thus increase livestock productivity (Dong et al., 2003). However, continuous cultivation of alfalfa has led to an increase in pathogenic bacteria and soil acidification, and these changes have been shown to be closely linked to soil microorganisms (Yan et al., 2012; Yao et al., 2019; Liu et al., 2020).

Previous research has shown that the length of the growth stage of alfalfa is related to grass productivity. In the arid regions of northeast China, alfalfa productivity increases for some years after planting, but decreases if planted for too long (Li and Huang, 2008). The ideal duration of the alfalfa growing stage was indicated to be 9 years (Jiang et al., 2007). Moreover, these studies have shown that changes in soil properties with the increasing years of alfalfa cultivation are significantly associated with alfalfa yield (Ren et al., 2011). Soil productivity and environmental safety could be directly or indirectly influenced by soil characteristics (Doran and Parkin, 1994; Yao et al., 2019; Wang et al., 2019; Shi et al., 2020). Luo et al. (2018) showed that growing alfalfa significantly increased nutrient content in the soil, such as SOM, TN, AP, and AK. Dong et al. (2016) observed that the levels of SOM, TN, and TP were considerably enhanced from native sandy grassland when new alfalfa fields were recovered. However, the effect of planting alfalfa on the soil was influenced by the year of successive plantings, with researchers finding a decreasing trend in soil nutrients within 10 years of successive alfalfa plantings, and the opposite trend after 10 years (Jiang et al., 2007).

Soil bacteria play an important role in maintaining plant health and rapidly respond to alterations in soil characteristics, as they can participate in nutrient cycling and influence the immune system of plants (Song et al., 2021). Microbial community structures vary significantly among different cropping systems (Zhou et al., 2018; Liu et al., 2019; Yao et al., 2019; Yuan et al., 2021) and depend on crop species, soil types, and type of cropping system. For instance, soil basal respiration and microbial biomass had progressively fallen from 3 to 9 years of continuous alfalfa cultivation, while it increased between 15 and 25 years (Jiang et al., 2007). Another study showed that continuous cropping of alfalfa considerably improved the microbial diversity and affected the microbial community assembly *via* the changed soil characteristics (Luo et al., 2018). Moreover, Yao et al. (2019) found that the abundance of *Paecilomyces* and *Phaeomycocentrospora* grew significantly with the continuous growth period of the alfalfa. The decreased abundance of *Pencillium* sp. and the increased abundance of *Fusarium* sp. were also found in the continuous copping of alfalfa (Xu et al., 1995). Nevertheless, it has also been shown that continuous cropping did not change the structure of the soil microbial community compared to crop rotation (Hu and Wang, 1996). The inconsistent results of the above studies may be due to differences in soil types, sampling and sequencing methods, crop rotation systems, and duration of continuous cultivation. Therefore, more in-depth studies in different farming systems and environments are needed to explore the mechanisms of barriers to continue cropping.

In view of the changing soil microorganisms to promote sustainable animal feed industry in northeast China, it is meaningful to study the changes among different cropping systems, i.e., meadow, maize, and alfalfa, and to reveal the alfalfa growing responses to long continuous cropping for 6, 10, 14, 20, and 30 years in their soil microorganisms and soil quality. The study examined soil samples from three agricultural systems and different years of continuously crop-grown alfalfa fields in northeast China, and evaluated the soil bacterial structure and soil characteristics. This study aimed to explore the soil microbial community structure of diverse crop systems and alfalfa with continuous cropping time and to assess the complete connection between soil bacterial communities and physical and chemical characteristics.

## Materials and Methods

### Experimental Site and Design

The experimental location is in Furalji District, Qiqihar City, Heilongjiang Province, China (4715′N, 12341′E). Fields that continuously planted alfalfa for 6, 10, 14, 20, and 30 years were selected and coded as C6, C10, C14, C20, and C30, respectively. Moreover, the soils of the meadows and maize field were selected as the controls, which encoded Me and Ma, respectively. Each treatment is over 900 m^2^ in size. The sowing density of alfalfa is 4,000,000 seeds ha^–1^. The chemical compound fertilizer (N 18%, P_2_O_5_ 18%, and K_2_O 18%) of 280 kg/ha was applied to each treatment in June each year. The fields of alfalfa are maintained using standard planting and are not greased. The alfalfa was cut to the surface in June and August each year, except for the first year when it is sown.

### Soil Sampling and Soil Characteristic Measurement

On June 30, 2019, during the flowering time of alfalfa, soil samples were collected at 0–15 cm ground depth. A combination of over five individual soil nuclei from a total area of 500 m^2^ was obtained from each sample. A total of 42 soil samples of meadow, maize, and 5 alfalfa fields were collected. Filtering of stones and plant roots from the soil sample was carried out using a 2-mm sieve. Approximately 2 *g* of soil samples was placed in sterilized centrifuge tubes and stored at −80°C in a refrigerator for soil DNA extraction; fresh soil was used to measure soil Physicochemical properties.

Using a pH meter, the soil pH was determined in a soil water suspension (1:5 w/v). Fifteen grams of fresh soil was dried in an oven at 105°C for 24 h to a constant weight to determine the soil moisture content. An elemental analyzer was used to measure the soil total nitrogen and carbon contents (Jones and Willett, 2006). Using the continuous flow analysis system, 2.0 M KCl was used to extract ammonium (NH_4_^+^–N) and nitrate (NO_3_^—^N). Moreover, 0.5 M NaHCO_3_ and H_2_SO_4_–HClO_4_ were used to extract the total and available phosphorus, respectively. Additionally, using an inductively coupled plasma emission spectrometry (ICPS-7500), HNO_3_–HClO_4_–HF and 1.0 M CH_3_COONH_4_ were used to extract soil total and available potassium, respectively (Lu, 1999).

### DNA Extraction and High-Throughput Sequencing

Using the Fast DNA Spin Kit (MP Biomedicals, Santa Ana, CA, United States), soil total DNA was extracted from soil samples. Primers of 515F/806R were used to amplify the bacterial 16S rRNA gene (White et al., 1990), and the forward primer being modified at the 5′ end with a unique 6-nt barcode was added. A 20-ml PCR mix with 0.5 ml of each 10 mM primer, 10 ng of DNA template, and 18 ml of Platinum PCR SuperMix were used to produce PCR. The PCR procedure was 95°C for 5 min; 94°C for 35 s, 55°C for 15 s, 72°C for 10 s for 32 cycles, and 75°C extension for 8 min (Liu et al., 2015). All the samples were standardized to equimolar levels and sequenced on the Majorbio Biotechnology Illumina MiSeq platform. All sequences are deposited in GenBank of NCBI with the archive PRJNA760979.

The raw FASTQ data were processed with QIIME Pipeline version 1.19.1 after sequencing. In brief, each barcode-based sample was allocated to all sequence reads. Preliminary analyses were performed to eliminate sequences of low quality (length < 200 bp and average basis quality score < 30). Use the UCHIME algorithm to find and eliminate chimera of the trimmed sequences (Edgar et al., 2011). The RDP classification was used to allocate sequences phylogenetically based on their optimal match to the RDP databases. Operational taxonomic units (OTUs) with a CD-HIT sequence similarity of 97% were categorized (Cole et al., 2009; Li and Godzik, 2015).

For the alpha diversity, Shannon and Chao 1 indices were calculated in QIIME. Additionally, principal coordinate analysis, Adonis test, and canonical correspondence analysis have been carried out in R version 4.1.1 with the “vegan” package. GenStat 13 was used to perform the one-way analysis of variance (ANOVA) to assess differences in soil chemistry and the abundance of bacteria at different taxonomic levels. Bacterial symbiotic networks were analyzed for the Me, Ma, and AC treatments, and AC6-10, AC14-20, and AC30 groups. The raw data were statistically analyzed using the “psych” package in R and then visualized in Gephi (Jiang et al., 2017). The correlation between each of the two OTUs was chosen to be *p* < 0.05, with Spearman correlation coefficients greater than 0.7 (Mendes et al., 2018). Identification of keystone species was based on high nodality, high intermediate centrality, and high compact centrality (Berry and Widder, 2014; Agler et al., 2016).

## Results

### Soil Physicochemical Characters

Soil pH, NO_3_^–^, TK, AK, and C/N were significantly higher in the alfalfa soils, compared with the soil of meadow and maize, while NH_4_^+^, TP, AP, and TN showed the opposite trend. Moreover, soil NO_3_^–^, AK, TC, and TN contents increased with the extension time in the soil of continuous crop alfalfa, whereas the contents of NH_4_^+^, TP, and AP decreased with the extension time in continuous cropping alfalfa soils (Table 1).

**TABLE 1:** Soil physicochemical properties of meadow, maize, and different years of alfalfa continuous cropping.

Treatment	pH	NH4	NO3	TP	TK	AK	AP	TC	TN	C/N
Me	5.66 ± 0.04d	2.24 ± 0.11a	0.48 ± 0.02d	0.66 ± 0.03a	19.97 ± 1.51d	128.5 ± 9.46d	40.5 ± 1.37a	27.75 ± 0.95a	2.72 ± 0.08a	10.22 ± 0.47d
Ma	7.6 ± 0.09c	2.18 ± 0.07a	1.76 ± 0.36a	0.62 ± 0.06a	21.97 ± 1.43c	249.7 ± 10.99a	38.72 ± 1.09b	17.9 ± 0.78d	1.72 ± 0.04c	10.38 ± 0.37d
AC6	7.8 ± 0.02ab	2.29 ± 0.19a	1.39 ± 0.07b	0.63 ± 0.03a	21.25 ± 0.49c	158.9 ± 4.57c	31.66 ± 1.11c	19.91 ± 0.74c	1.55 ± 0.07d	12.87 ± 0.29a
AC10	7.75 ± 0.06b	1.83 ± 0.06c	1.19 ± 0.08c	0.47 ± 0.06b	25.42 ± 0.57a	151.9 ± 8.78c	28.4 ± 1.78d	19.65 ± 0.88c	1.68 ± 0.09c	11.73 ± 0.82c
AC14	7.81 ± 0.04ab	2 ± 0.05b	1.29 ± 0.05bc	0.22 ± 0.01c	24.61 ± 0.75ab	168.8 ± 7.48b	11.71 ± 1.15e	21.34 ± 0.89b	1.77 ± 0.09c	12.05 ± 0.52bc
AC20	7.83 ± 0.06a	1.87 ± 0.03c	1.41 ± 0.08b	0.25 ± 0.02c	24.59 ± 0.51ab	150 ± 5.2c	9.61 ± 0.18f	21.74 ± 0.59b	1.75 ± 0.09c	12.45 ± 0.56ab
AC30	7.76 ± 0.03ab	1.88 ± 0.05c	1.75 ± 0.11a	0.14 ± 0.03d	23.96 ± 0.76b	170.2 ± 9.78b	9.02 ± 0.13f	22.31 ± 0.74b	1.92 ± 0.08b	11.6 ± 0.24c

### Soil Bacterial Diversity

**FIGURE 1:**
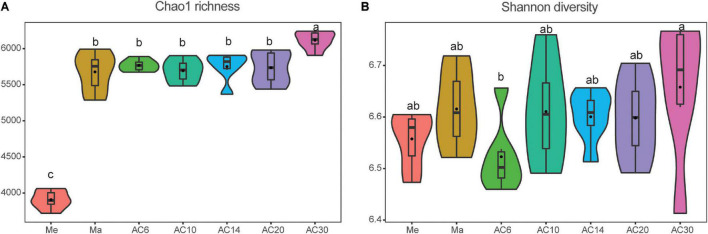
Effect of crop type and continuous cropping years on the bacterial Chao1 richness **(A)** and Shannon diversity **(B)**.

**FIGURE 2:**
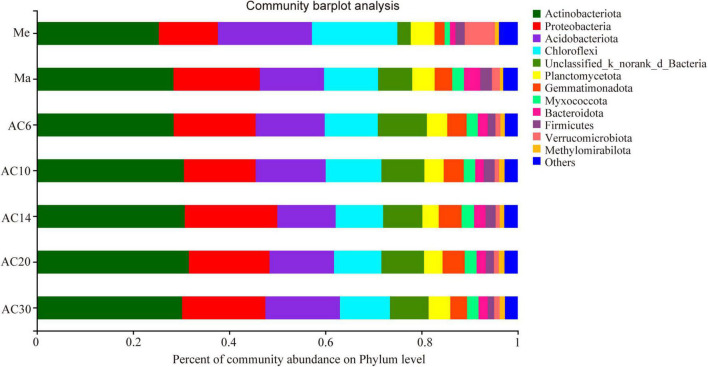
Effect of crop type and continuous cropping years on the bacterial phylum.

**FIGURE 3:**
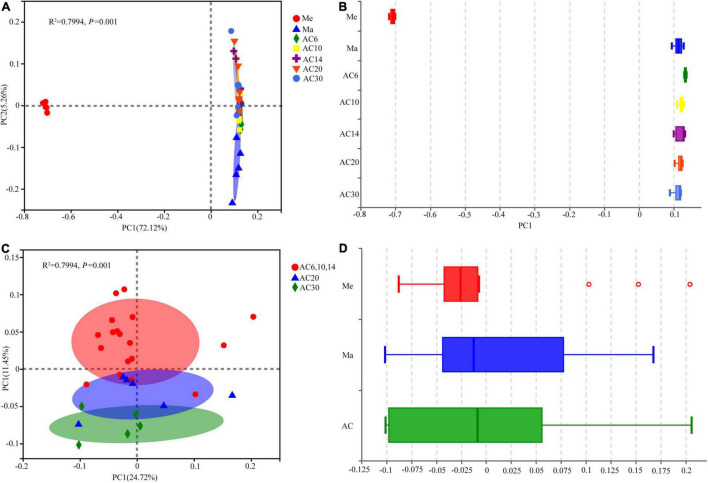
Principal coordinate analysis (PCoA) based on Bray–Curtis dissimilarities in the soil among the seven treatments **(A,B)** and among alfalfa continuous cropping different years **(C,D)**.

**FIGURE 4:**
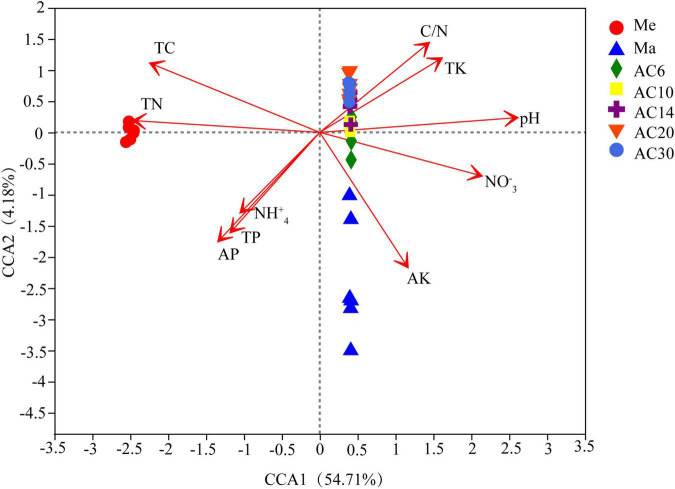
Canonical correspondence analysis (CCA) of bacterial communities changes with the environmental variables.

According to the Chao index, soil microbial diversity was highest in the AC30 treatment and lowest in the Me treatment (*p* > 0.05; Figures 1A,B). Effect of crop type and continuous cropping years on the bacterial phylum (Figure 2). Principal coordinate analysis (PCoA) revealed that cropping systems and alfalfa continuous cropping time significantly affected the soil bacterial communities (PERMANOVA, *p* < 0.05; Figures 3A–D and Table 2). According to the PCoA result, we divided all treatments into three groups—Me (Meadow), Ma (Maize), and AC (Alfalfa continuous cropping)—and further divided AC into three groups—AC6-10 (alfalfa continuous cropping for 6, 10, and 14 years), AC20 (alfalfa continuous cropping for 20 years), and AC30 (alfalfa continuous cropping for 30 years) (Figure 3 and Table 2). The results of CCA revealed that there was a close relationship between soil physicochemical and soil bacterial community composition (Figure 4). Specifically, total C (*r* = 0.764; *p* < 0.01) and N (*r* = 0.654; *p* < 0.01), C/N (*r* = 0.876; *p* < 0.01), TP (*r* = 0.732; *p* < 0.05), AK (*r* = 0.732; *p* < 0.01) and TK (*r* = 0.804; *p* < 0.01), NH_4_ (*r* = 0.677; *p* < 0.05), pH (*r* = 0.616; *p* < 0.01), and NO_3_ (*r* = 0.677; *p* < 0.05) seemed significantly associated with the microbial community composition.

**TABLE 2:** Effect of crop type and continuous cropping years on the differences of bacteria communities based on PERMANOVA analysis.

Pairwise comparison	*R*	*p*
Me vs. Ma	0.865	0.001***
Me vs. AC	0.885	0.001***
Me vs. AC	0.839	0.002
AC6,10,14 vs. AC20	0.645	0.017
AC6,10,14 vs. AC30	0.768	0.001***
AC20 vs. AC30	0.654	0.003

***Significant P-value of 0.01.

### Specific Microbiomes

**FIGURE 5:**
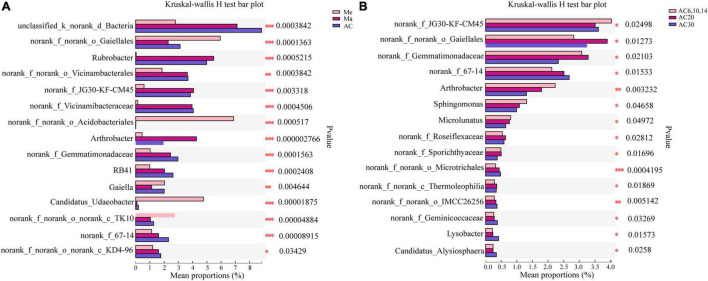
Effect of crop type **(A)** and continuous cropping years **(B)** on the differentiation of bacterial genus.

Actinobacteria, Acidobacteria, Proteobacteria, and Chloroflexi were the phyla with the highest relative abundance across all the treatments, accounting for 72.14–78.81% of the whole community (Figure 3). Overall, the relative abundance of Actinobacteria and Proteobacteria was higher in the Ma and AC treatments compared with the Me treatment, while Acidobacteria showed the opposite trend. On the genera level, the relative abundance with Kruskal–Wallis *H* test showed that some genera, such as *norank_Gaiellales*, *norank_Vicinambacterales*, *Rubrobacter*, and *Arthrobacter*, were significantly (*p* < 0.05) different among the Me, Ma, and AC fields. Moreover, some genera, such as *norank_JG30-KF-CM45, norank_Gaiellales, Arthrobacter, Sphingomonas, Microlunatus*, and *Lysobacter*, were significantly (*p* < 0.05) different among the cropping systems of alfalfa continuous cropping for AC6-10, AC20, and AC30 treatments (Figure 5). In more detail, the relative abundance of *Rubrobacter*, *norank_Vicinambacterales*, *norank_JG30-KF-CM45, norank_Vicinamibacteraceae*, *Arthrobacter*, and *norank_Gemmatimonadaceae* was significantly higher in the Ma and AC treatments compared with the Me treatment, while the relative abundance of *norank_Acidobacteriales*, *Candidatus_Udaeobacter*, and *norank_TK10* showed the opposite trend (Figure 5A). Furthermore, the relative abundance of *norank_Gaiellales*, *norank_67-14*, *norank_Gemmatimonadaceae*, and *Lysobacter* were increased with the alfalfa continuous cropping time, while *norank_JG30-KF-CM45*, *Arthrobacter*, *norank_Geminicoccaceae*, *Sphingomonas*, and *Microlunatus* showed the opposite trend (Figure 5B).

### Co-occurrence Network

**FIGURE 6:**
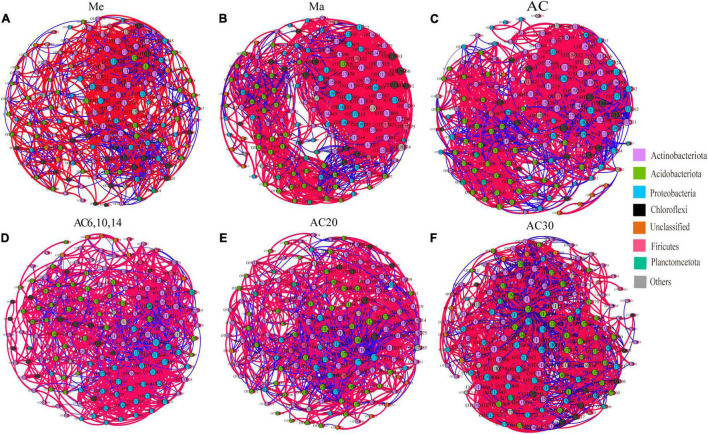
Co-occurrence network of the soil bacterial community for Me **(A)**, Ma **(B)**, AC **(C)**, AC6, 10, 14 **(D)**, AC20 **(E)** and AC30 **(F)** treatment.

The co-occurrence network based on OTU level shows the relationship between bacteria in different treatments (Figure 6). Comparing the Me, Ma, and AC treatments, the ranking of the number of negative correlations and modularity was Me > AC > Ma, while for the average degree (avgK) and clustering coefficient (avgCC), no significant differences were found among the treatments. When comparing the AC6-10, AC20, and AC30 treatments, the number of negative correlations, modularity, and avgCC increased with the years of continuous cropping. For the keystone species, OTU1210 (*Jatrophihabitans*), OTU10961 (*Blastococcus*), and OTU8174 (*norank_Gemmatimonadaceae*) were identified in the Me, Ma, and AC networks, respectively, while OTU13196 (*Microlunatus*), OTU5705 (*Paenibacillus*), and OTU8419 (*norank_Xanthobacteraceae*) were identified in the AC6-10, AC20, and AC30 networks, respectively (Table 3).

**TABLE 3:** Topological characteristics of soil bacterial co-occurrence network.

Network metrics	Me	Ma	AC	AC6,10,14	AC20	AC30
Number of nodes	150	150	150	150	150	150
Number of edges	1,338	2,089	1,951	1,416	1,601	2,023
Number of positive correlations	953	1,748	1,582	1,054	1,141	1,348
Number of negative correlations	385	340	369	362	460	675
Average degree (avgK)	17.96	27.853	26.013	18.88	21.347	26.973
Average weighted degree	7.638	23.417	17.018	11.617	8.77	9.339
Network diameter	7	9	8	8	6	7
Graph density	0.121	0.187	0.175	0.127	0.143	0.181
Modularity (M)	1.046	0.587	0.626	1.131	1.099	1.52
Interconnecting piece	1	3	3	2	1	2
Average clustering coefficient (avgCC)	0.615	0.694	0.699	0.591	0.631	0.662
Average path length (APL)	2.855	2.984	2.689	2.63	2.69	2.546

**TABLE 4:** Keystone taxa identified in the co-occurrence network.

	OTU ID	Phylum	Class	Order	Family	Genus	Species
Me	OTU1210	Actinobacteriota	Actinobacteria	Frankiales	Frankiaceae	*Jatrophihabitans*	*norank*
	OTU1028	Proteobacteria	Gammaproteobacteria	Burkholderiales	Rhodocyclaceae	*norank*	*norank*
	OTU1098	Actinobacteriota	Actinobacteria	Frankiales	norank	*norank*	*norank*
Ma	OTU10961	Actinobacteriota	Actinobacteria	Frankiales	Geodermatophilaceae	*Blastococcus*	*norank*
	OTU11804	Acidobacteriota	Blastocatellia	Blastocatellales	Blastocatellaceae	*norank*	*norank*
	OTU6973	Proteobacteria	Alphaproteobacteria	Rhizobiales	Rhizobiaceae	*Allorhizobium*	*Pararhizobium*
AC	OTU8174	Gemmatimonadota	Gemmatimonadetes	Gemmatimonadales	Gemmatimonadaceae	*norank*	*norank*
	OTU10318	Actinobacteriota	Thermoleophilia	Gaiellales	norank	*norank*	*norank*
	OTU5813	Chloroflexi	Chloroflexia	Thermomicrobiales	JG30-KF-CM45	*norank*	*norank*
AC6,10,14	OTU13196	Actinobacteriota	Actinobacteria	Propionibacteriales	Propionibacteriaceae	*Microlunatus*	*norank*
	OTU8174	Gemmatimonadota	Gemmatimonadetes	Gemmatimonadales	Gemmatimonadaceae	*norank*	*norank*
	OTU12811	Acidobacteriota	Vicinamibacteria	Vicinamibacterales	Vicinamibacteraceae	*norank*	*norank*
AC20	OTU5705	Firmicutes	Bacilli	Paenibacillales	Paenibacillaceae	*Paenibacillus*	*Paenibacillus*
	OTU12040	Proteobacteria	Gammaproteobacteria	Burkholderiales	Comamonadaceae	*norank*	*norank*
	OTU11037	Actinobacteriota	Actinobacteria	Micrococcales	Microbacteriaceae	*Agromyces*	*Agromyces*
AC30	OTU8419	Proteobacteria	Alphaproteobacteria	Rhizobiales	Xanthobacteraceae	*norank*	*norank*
	OTU11007	Actinobacteriota	Thermoleophilia	norank	norank	*norank*	*norank*
	OTU9218	Proteobacteria	Gammaproteobacteria	Burkholderiales	SC-I-84	*norank*	*norank*

## Discussion

In the present study, the Ma and AC treatments have higher microbial diversity than the Me treatment, and microbial diversities increased significantly in the long-term continuous (AC30) treatment. These results suggest that maize and alfalfa were enriched with more microbial species and were more conducive to soil conservation and sustainability, at least in terms of microbial diversity. Previous studies have found that, compared with corn–soybean rotation systems, there was less rhizosphere bacterial diversity in continuously grown soybeans (Liu et al., 2020). A positive correlation between continuous cropping years and soil bacterial diversity has also been reported (Liu et al., 2020). Nevertheless, it has also been claimed that soil microbial diversity did not differ between soils grown in continuous soybean and soybean–maize rotations (Li et al., 2010). The different results of these studies might depend on the types of soil utilized and the different years of continuous cropping. Furthermore, differences in crop genotypes may also be responsible for this phenomenon, as microbial diversity has also shown different trends due to successive plantings of resistant and sensitive varieties (Yuan et al., 2021). Changes in soil pH can affect other soil physicochemical properties, and these changes directly or indirectly influence microbial diversity (Tan et al., 2017; Lian et al., 2019). In addition, microbial diversity in different farming systems can be affected by changes in plant root secretions, such as flavonoids and hormones (Tan et al., 2017; Lian et al., 2019; Liu et al., 2020; Shi et al., 2020).

From the results of PCoA and the PERMANOVA analysis, the crop types and years of continuous alfalfa were considered the two most important factors that changed the soil bacterial structure (*p* < 0.05). There is no doubt that different crops have different microbial community structures (Lian et al., 2019). This was in line with some previous studies that have shown significant variation in soil bacterial communities in short- and long-term alfalfa continuous cropping field (Zhu et al., 2017; Yao et al., 2019). The CCA result demonstrated that the major factors in changing soil bacterial community structure in different treatments in this study were soil pH, NO_3_^–^, total K, total P, and available P. Similar results were found for the significant effect of soil characteristics, such as soil pH and AP, on the structure of the bacterial community. In our investigation, these soil parameters were impacted significantly by continuous cropping, showing that continuous crops modified their soil characteristics and subsequently changed their bacterial community.

In the Ma and AC soils compared with those of the Me system, the relative abundance of Actinobacteria and Proteobacteria was substantially enhanced, suggesting that the bacteria were increased with high nutrient availability (Li et al., 2014; Yuan et al., 2021). The relative abundances of *Arthrobacter* increased in the Ma and AC cropping field compared with Me, but then decreased in the AC20 and AC30 long continuous cropping field, compared with AC6-14. Hexavalent chromium can cause serious human irritation, while *Arthrobacter* can reduce hexavalent chromium, thus making the soil environment more beneficial. Some specific metabolites of *Arthrobacter* can promote amino acid secretion from plant roots (Romaniuk et al., 2018; Shi et al., 2020). Additionally, some microbial species, such as *norank_Gaiellales* and *Lysobacter*, which play a role in ecological function of ligninolysis and in soil suppression against the fungal root pathogen, were increased with the alfalfa continuous cropping time, suggesting that these bacteria might inhibit soil fungal diseases due to long-term continuous cropping (Gómez Expósito et al., 2015). Therefore, changes in these bacteria across treatments may be related to antagonistic activity of plant pathogens and improved soil nutrition. However, the contribution of these significantly responsive microbial species to the plant is speculative based on their abundance and reported function. Whether they have a definite role in continuous cropping for alfalfa requires further verification.

Association network analysis provides a more detailed understanding of bacterial community composition and associations (Xue et al., 2018; Xiong et al., 2021). The network negative correlations and modularity of the Me were higher than that in Ma and AC treatments, suggesting that continuous planting of alfalfa promotes cooperation between bacteria, which facilitates the long growth of alfalfa and is beneficial to the soil (Yao et al., 2019). This finding corresponds to an earlier research, showing that the soil microbial structure becomes increasingly healthy after a long period of continuous cropping (Yao et al., 2019; Liu et al., 2020).

In summary, maize cropping and continuous cropping of alfalfa increased the soil bacterial alpha diversity, and alpha diversity also increased in the long-term continuous planting system. Soil pH, NO_3_^–^, total K, and total P content were important factors influencing the structure of soil bacterial community in different plant types and different alfalfa continuous cropping system. Moreover, compared with planting meadow, maize and alfalfa continuous cropping significantly increases a number of beneficial bacterial species, such as *Arthrobacter* and *Gaiellales*, suggesting that the microbial community of maize and long-term alfalfa cropping shifts toward a healthy pattern. However, these microorganisms need to be isolated and formed into synthesized microbial communities to verify their specific benefits to the crop. Furthermore, the networks differ among different plant types and also differ among different continuous cropping years of alfalfa. The topology of the networks suggested that continuous planting of alfalfa promotes cooperation between bacteria, which facilitates the long growth of alfalfa and is beneficial to the soil.

## Data Availability Statement

The datasets presented in this study can be found in online repositories. The names of the repository/repositories and accession number(s) can be found in the article/supplementary material.

## Author Contributions

HL and ZY conceived and designed this study. ZY and YX performed the experiments and wrote the manuscript. SL, XW, and HC analyzed the data. All authors approved the final version of the manuscript.

## Conflict of Interest

The authors declare that the research was conducted in the absence of any commercial or financial relationships that could be construed as a potential conflict of interest.

## Publisher’s Note

All claims expressed in this article are solely those of the authors and do not necessarily represent those of their affiliated organizations, or those of the publisher, the editors and the reviewers. Any product that may be evaluated in this article, or claim that may be made by its manufacturer, is not guaranteed or endorsed by the publisher.
